# Suppression of p53 by Notch3 is mediated by Cyclin G1 and sustained by MDM2 and miR-221 axis in hepatocellular carcinoma

**DOI:** 10.18632/oncotarget.2523

**Published:** 2014-09-25

**Authors:** Catia Giovannini, Manuela Minguzzi, Michele Baglioni, Francesca Fornari, Ferdinando Giannone, Matteo Ravaioli, Matteo Cescon, Pasquale Chieco, Luigi Bolondi, Laura Gramantieri

**Affiliations:** ^1^ Center for Applied Biomedical Research (CRBA), S.Orsola-Malpighi University Hospital, Bologna, Italy; ^2^ Department of Medical and Surgical Sciences University of Bologna, Bologna, Italy; ^3^ Department of Medical and Surgical Sciences, General and Transplant Surgery Unit, University of Bologna, Bologna, Italy

**Keywords:** Notch3, p53, miR-221, CyclinG1, MDM2

## Abstract

To successfully target Notch receptors as part of a multidrug anticancer strategy, it will be essential to fully characterize the factors that are modulated by Notch signaling. We recently reported that Notch3 silencing in HCC results in p53 up-regulation *in vitro* and, therefore, we focused on the mechanisms that associate Notch3 to p53 protein expression. We explored the regulation of p53 by Notch3 signalling in three HCC cell lines HepG2, SNU398 and Hep3B.We found that Notch3 regulates p53 at post-transcriptional level controlling both Cyclin G1 expression and the feed-forward circuit involving p53, miR-221 and MDM2. Moreover, our results were validated in human HCCs and in a rat model of HCC treated with Notch3 siRNAs. Our findings are becoming an exciting area for further in-depth research toward targeted inactivation of Notch3 receptor as a novel therapeutic approach for increasing the drug-sensitivity, and thereby improving the treatment outcome of patients affected by HCC. Indeed, we proved that Notch3 silencing strongly increases the effects of Nutilin-3. With regard to therapeutic implications, Notch3-specific drugs could represent a valuable strategy to limit Notch signaling in the context of hepatocellular carcinoma over-expressing this receptor.

## INTRODUCTION

Understanding the complexity of cancer depends on an elucidation of the underlying regulatory networks, at the cellular and intercellular levels [1]. On this regard Notch receptors have been extensively studied in the last decade and numerous pathways that crosstalk with Notch signaling have been described [2]. Thus, therapeutic targeting of the Notch signaling presents both promise and challenges. The successful development of a Notch based targeted cancer therapy, however, will require a better elucidation of the underlying cellular and intercellular regulatory networks associated with the activation of this evolutionary conserved family of receptors.

The Notch3 receptor is highly expressed in nearly 80% of hepatocellular carcinomas (HCCs) but is barely detectable in surrounding cirrhotic tissue and in normal liver [3]. Our previous study demonstrated that a stable Notch3 silencing increases the levels of p53 protein in HCC cell lines. However, the underlying molecular mechanisms remain to be clarified. The tumour suppressor p53 is a powerful anti-tumoral molecule frequently inactivated by mutations or deletions in cancer. Nevertheless, half of all human tumours express wild-type p53, and its activation by antagonizing the effects of its negative regulators might offer a new therapeutic strategy [4]. P53 is regulated by complex networks of translational and post-translational modifications, including phosphorylation, ubiquitination, and proteosome degradation. The MDM2 (mouse double minute protein 2) gene is induced by p53 and the protein binds the transcriptional activation domain of p53, blocking the recruitment of factors necessary for induction of gene expression [5]. Moreover MDM2 targets p53 for degradation by acting as an E3 ubiquitin ligase [6]. Thus, p53 and MDM2 form an auto-regulatory loop in which p53 positively regulates MDM2 expression and MDM2 negatively regulates p53 [7]. The cell cycle regulator Cyclin G1 was identified as a homologous protein to c-src and, later, it was found to be a target gene of p53 [8, 9]. However the transcription of Cyclin G1 can be regulated by p53-dependent and independent pathways [10]. Cyclin G1 negatively affects the stabilization of p53 by promoting protein degradation through a negative feedback signaling to the p53-MDM2 auto-regulatory module [11]. One function of Cyclin G1 is to modulate the phosphorylation of MDM2 and thereby its regulation of p53. Indeed the phosphorylation of MDM2 Thr216 residue was found to greatly increase in Cyclin G^−/−^ cells compared to wild-type MEFs leading to higher levels of p53 protein [12]. These findings suggest that both Cyclin G1 and MDM2 are master regulators of the p53 protein. Here we show that p53 up-regulation in Notch3 silenced cells is first mediated by Cyclin G1 down-regulation and than sustained by decreased level of MDM2 due to miR-221 up-regulation by p53.

## RESULTS

### Notch3 regulates p53 at post-transcriptional level

**Figure 1:**
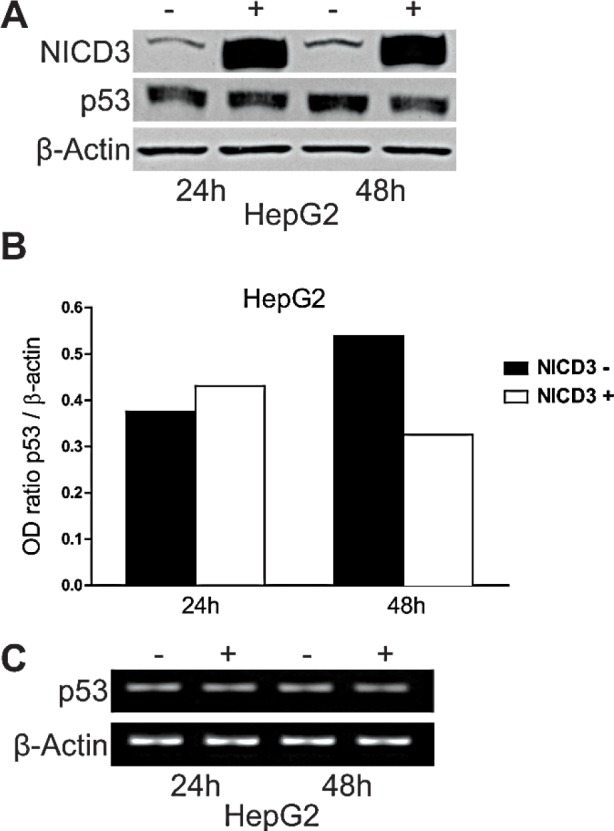
Effects of Notch3 over-expression on p53 levels HepG2 cells were transfected with pNICD-3 expression vector and p53 was evaluated both at protein and mRNA level 24h and 48h post-transfection (A,C). B) The level of p53 protein showed in panel A was quantified and expressed as a ratio with respect to β actin control. β Actin was used as a reference control for both protein and mRNA levels. (−): cell transfected with empty vector; (+):cells transfected with NICD3.

We previously showed that Notch3 knock-down in HepG2 and SNU398 cell lines results in the accumulation of p53 that exacerbated their sensitivity to doxorubicin by inducing apoptosis. Herein we extended our study to understand whether Notch3 regulates p53 at transcriptional or post-transcriptional level. P53 mRNA levels were not altered in Notch3 silenced cells as detected by Real Time-PCR (data not shown). To further confirm the role of Notch3 in p53 protein regulation, HepG2 cells were transfected with human Notch3 ICN (Notch Intracellular Domain) or vector alone (pcDNA3) and both p53 mRNA and protein levels were analyzed. Plasmids were cross-linked with a green fluorophore and sorted to analyse only transfected cells. We used Western blot analysis to confirm the over-expression of Notch3 in Notch3 ICN-transfected cells and we found a higher level of Notch3 expression (Fig.1A), which resulted in reduced levels of p53 only at 48h post-NICD3 transfection (Fig.1A-B). The different effect observed at different time post- NICD3 transfection could be due to a different percentage of green cells sorted at 24h and 48h and/or it reflects the time due to NICD3 to exert its effects on p53. Conversely, Notch3 over-expression has no effect on p53 mRNA level (Fig.1C). Taken together our results suggest that Notch3 regulates p53 at post-transcriptional level.

### MDM2 protein expression is regulated by miR-221 in Notch3 depleted cells

**Figure 2:**
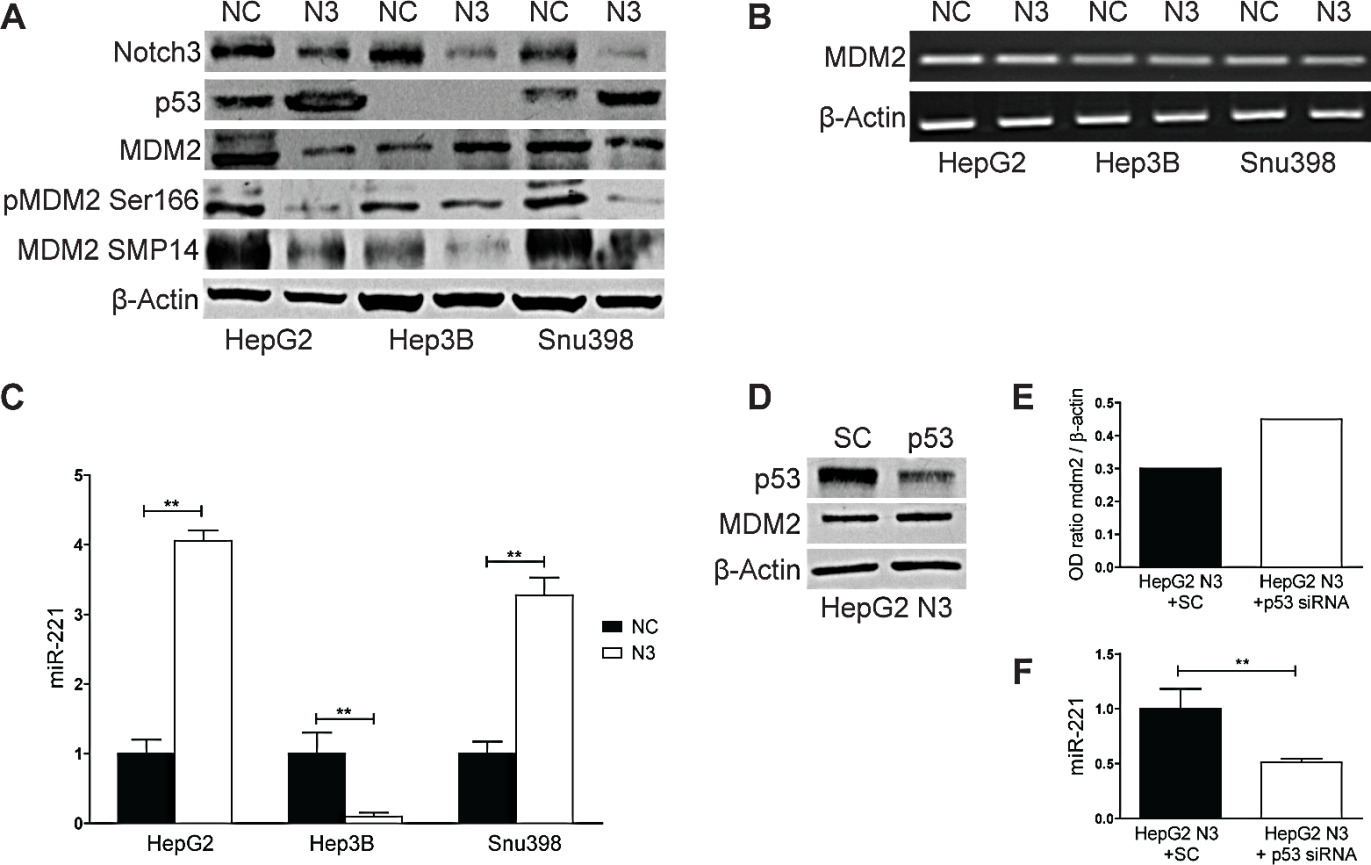
Notch3 controls MDM2 protein expression through miR221 A-B) Efficacy of Notch3 KD on MDM2 protein and mRNA expression was measured by western blotting and RT-PCR respectively in HepG2, Hep3B and SNU398 cells. P53 and MDM2 phosphorylation status of Ser166 and Thr216 were also evaluated. C) Real-Time PCR of miR-221 in Notch3 silenced cells. Results are the mean of three independent experiments (+/− S.E.). P values (by two tailed student's t test) were < 0.01 for N3 vs NC in all the analyzed cell lines. D) HepG2 Notch3 silenced cells were transfected with p53 siRNA or scrambled RNA and p53 knockdown and MDM2 protein levels were evaluated 48h post-transfection by western blot. E) The level of MDM2 protein showed in panel D was quantified and expressed as a ratio with respect to β actin control. F) Real-Time PCR of miR-221 in HepG2 Notch3 silenced cells transfected with p53 siRNA or scrambled RNA. Results are the mean of three independent experiments (+/− S.E.). **, P<0.01 (by two tailed student's t test). NC: negative control shRNA; N3; Notch3 shRNA; SC: scramble RNA; p53: p53 siRNA.

By promoting p53 ubiquitination and the consequent degradation the MDM2 protein is the primary regulator of p53 stability [13]. To investigate the involvement of MDM2 in the increased levels of p53 we assessed the expression levels of MDM2 in Notch3 depleted cells. As shown in Fig.2A, MDM2 protein expression was reduced in HepG2 and SNU398 cells whereas its expression was increased in Hep3B suggesting that MDM2 is not a direct effector of Notch3. No difference was observed in MDM2 mRNA expression in Notch3 silenced cells compared to negative control (Fig.2B). As it was previously proven in normal chondrocytes and in HCC cell lines MDM2 is a predicted target of miR-221 [14, 15]. This prompted us to investigate miR-221 expression in Notch3 depleted cells and we found that it was up-regulated in HepG2 and SNU398 and down-regulated in Hep3B (Fig.2C). Fornari et al. recently demonstrated that p53 triggers miR-221 transcription by binding its upstream region and that miR-221 up-regulation by p53 exerts a positive feed back loop by targeting MDM2 [15]. This observation let us to hypothesize that the high levels of p53 observed in Notch3 depleted cells could increase miR-221 that, in turn, decreases MDM2 levels. Indeed in Hep3B cells which are TP53−/− we observed reduced levels of miR-221 and increased levels of MDM2 in Notch3 silenced cells compared to control cells (Fig.2A, 2C). Moreover p53 silencing in Notch3 depleted cells resulted in miR-221 down-regulation and MDM2 up-regulation and (Fig.2D-F). These findings let us hypothesize the contribution of p53/miR-221 axis to MDM2. However, our results do not exclude a possible involvement of other factors, such as the different genetic background or the role of others microRNAs targeting MDM2.

### HES1 binds to miR-221 promoter

**Figure 3:**
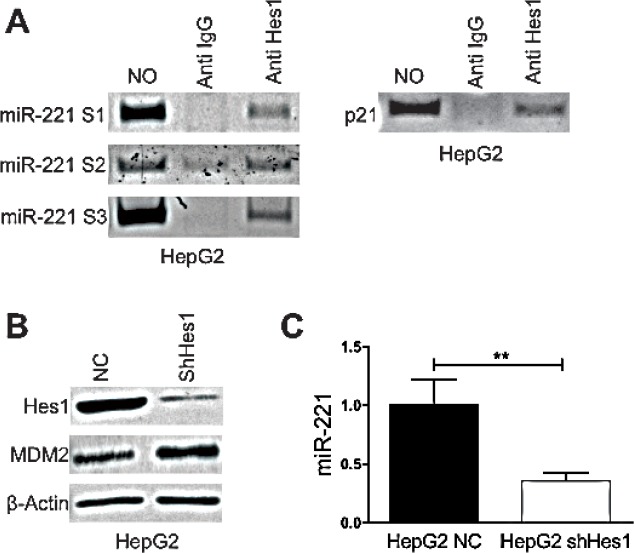
Hes1 regulates miR221 expression A) DNA samples of HepG2 cells was cross-linked with formaldehyde, and immunoprecipitated with anti-Hes1 or control rabbit immunoglobulin G (Cont. IgG). DNAs were extracted from the immunocomplexes and PCR amplified by the primer set of miR-221 promoter. DNA without ChIP served as a control (the first lane). P21 was used as positive control region. B) HepG2 cells were infected with Hes1 shRNA and Hes1 knockdown and MDM2 expression were evaluated by western blot. C) Real-Time PCR of miR-221 in Hes1 silenced cells. Results are the mean of three independent experiments (+/− S.E.). P values (by two tailed student's t test) was < 0.01 for shHes1 vs. NC in all the analyzed cell lines. NC: negative control shRNA.

As described above the knockdown of Notch3 in Hep3B cells reduced miR-221 expression leading us to hypothesize that a Notch3 target genes might affect miR-221 transcription. Hes1 is a basic helix-loop-helix (bHLH) type of transcriptional factor that regulates the expression of downstream target genes and is one of primary target of Notch signaling [16, 17]. We previously showed that Hes1 protein expression was diminished by Notch3 knock-down [18]. A bioinformatic analysis was executed in a region spanning −2500 + 1100 bp considering +1 the first nucleotide of miR-221 precursor. Three Hes1 consensus sequences, S1 (+521bp), S2 (−1827 bp), S3 (−2267 bp) were identified. To confirm the binding of Hes1 to miR-221 promoter a ChIP assay was performed as previously describes [19]. In HepG2 cells DNA of the miR-221 promoter region could be specifically detected in the Hes1-immunoprecipitated DNA complex from formaldehyde-treated cells, indicating Hes1 occupancy at the miR-221 promoter (Fig.3A). Hes1 silencing with specific shRNAs, by stable retroviral transduction (Fig.3B) determined a reduction of miR-221 expression and increased levels of MDM2 (Fig.3B-C). These results collectively show that Hes1 induces the transcription of miR-221 via direct binding to its promoter region.

### Notch3 regulates MDM2 phosphorylation at Ser166 and Thr216

The mechanisms herein described suggest that p53 increase consequent to Notch3 knockdown is sustained but not triggered by the p53-miR221 positive feedback loop. Therefore other mechanisms should be responsible for the p53 accumulation observed in Notch3 depleted cells. The first step in p53 stabilization is the phosphorylation of Ser20 and Ser15 that inhibits MDM2 binding [20, 21]. However we did not observe significant changes in p53 phosphorylation status between Notch3 silenced cells and negative control (Supplemental Fig.1A) [22]. The second step in p53 accumulation is dependent on the ability of MDM2 to mediate p53 degradation. More specifically MDM2 phosphorylation status regulates p53 stability. MDM2 phosphorylated at Thr216 binds less well to p53 leading to p53 accumulation [12]. Conversely MDM2 phosphorylation at Ser166 activates MDM2 resulting in diminished levels and decreased transcriptional activity of p53 [23]. When MDM2 is phosphorylated at Thr216 it loses reactivity with an MDM2-specific monoclonal antibody SMP14 [24]. As shown in Figure 2A, MDM2 phosphorylation on Thr216 and Ser166 resulted up-regulated and down-regulated after Notch3 knockdown in all the analyzed cell lines respectively. Our evidences suggest that, in the presence of functional p53, both reduced level of MDM2 protein expression and changes in phosphorylation status might be responsible for sustained p53 levels in Notch3 silenced cells.

### P53 accumulation in Notch3 depleted cells is triggered by Cyclin G1

**Figure 4:**
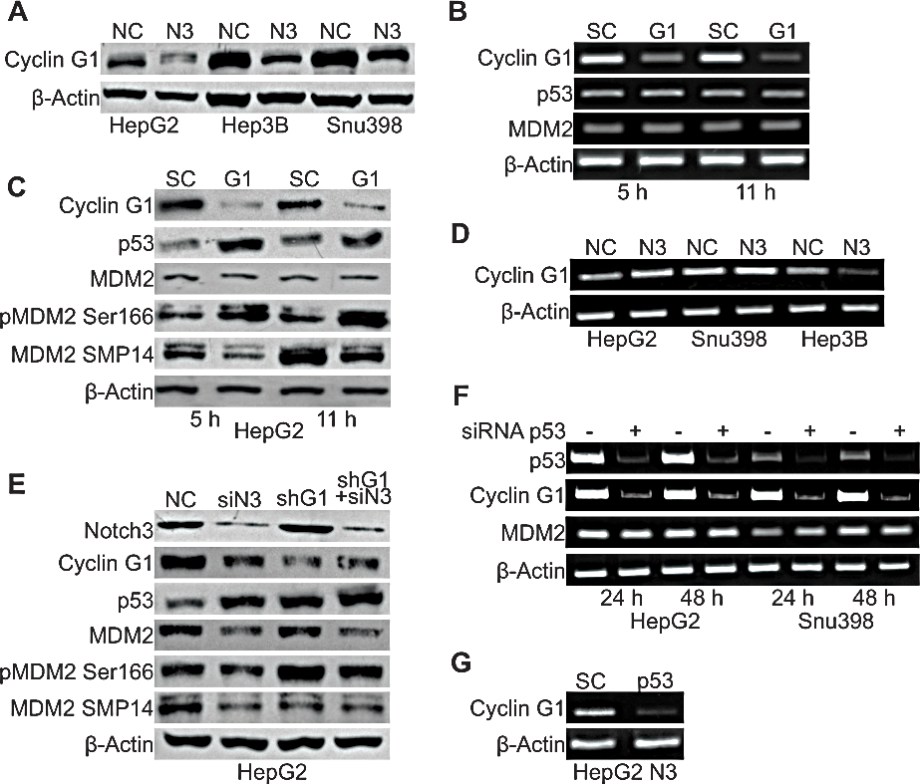
Cyclin G1 regulates p53 accumulation in Notch3 depleted cells A) Efficacy of Notch3 KD on Cyclin G1 protein expression was measured by western blotting in HepG2, Hep3B and SNU398 cells. B-C) HepG2 cells were transiently transfected with a pool of siRNAs directed against Cyclin G1 or scramble RNA (SC) for 5h and 11h. The level of p53 and MDM2 expression was evaluated by RT-PCR and western-blot. MDM2 phosphorylation status at Ser166 and Thr216 was also evaluated by western blot in Cyclin G1 silenced cells. D) Cyclin G1 mRNA expression evaluated by RT-PCR in Notch3 KD cells. E) Efficacy of Cyclin G1 + Notch3 silencing on different proteins expression was measured by western blotting. F) Semi-quantitative RT-PCR expression analysis of Cyclin G1 and MDM2 in p53 silenced cells. G) HepG2 Notch3 silenced cells were transfected with p53 siRNA or scrambled RNA and Cyclin G1 mRNA levels were evaluated 48h post-transfection by RT-PCR. P53 silencing was verify by western blot as shown in Figure 2D. NC: negative control shRNA; N3; Notch3 shRNA; siN3: Notch3 siRNA; shG1: Cyclin G1 shRNA; SC: scramble RNA; G1: Cyclin G1 siRNA; p53: p53 siRNA.

To investigate the mechanisms associated with MDM2 functional modifications observed in Notch3 depleted cells, we examined the expression of Cyclin G1, an established regulator of MDM2 phosphorylation. It has been shown that Cyclin G1 plays a key role in the regulation of MDM2 phosphorylation *in vivo* with corresponding impact on p53 protein. Indeed MDM2 protein in Cyclin G1 knockout mice is hyper-phosphorylated at Thr216 and the levels of p53 are significantly higher than those in wild type mouse embryonic fibroblasts [12]. These observations suggest a possible involvement of Cyclin G1 in p53 up-regulation in Notch3 depleted cells. To test this hypothesis we analyzed Cyclin G1 protein expression and we found reduced levels in all the analyzed cell lines in the absence of Notch3 expression (Fig.4A). To determine if lower cyclin G1 levels were associated with higher levels of p53 shown by Notch3 KD cells, we ablated endogenous Cyclin G1 expression by transient siRNA transfection in HepG2 cells (Fig.4C). Cyclin G1 silencing increased p53 protein levels whereas p53 mRNA resulted unaffected (Fig.4B). Finally, we examined whether Cyclin G1 silencing modifies the phosphorylation status of MDM2 at Ser166 and Thr216. Figure 4C shows that SMP14 reactivity with MDM2 protein was reduced in Cyclin G1 silenced cells compared to negative control. Contrary, reactivity of MDM2 with anti-phospho S166 increased in the absence of cyclin G1. Moreover, total MDM2 protein expression resulted independent by Cycling G1. On the mRNA side, semi-quantitative RT-PCR analysis in Cyclin G1 silenced cells revealed unchanged levels of MDM2 (Fig.4B). To establish that the increase in p53 levels after Notch3 knockdown is dependent on Cyclin G1, we checked p53 protein levels in HepG2 Notch3 silenced cells and in HepG2 Cyclin G1+ Notch3 silenced cells. Notch3 depleted cells and double silenced cells (shG1 + siN3) showed comparable p53 protein levels suggesting that Cyclin G1 is responsible for the increased p53 protein levels in Notch3 silenced cells (Fig.4E). No difference were observed in total MDM2 and in MDM2 phosphorylation at Thr216 between Notch3 silenced cells and double silenced cells. As expected, the phosphorylation status of MDM2 at Ser166 does not change between double silenced and negative control cells since Notch3 and Cyclin G1 have opposite effects on this phosphorylation as above described (Fig.4E).

From the data it appears that Cyclin G1 might be one of the reason for p53 accumulation following Notch3 depletion through regulation of MDM2 phosphorylation at Thr216, presumably through its interaction with PP2A as previously demonstrated [12].

The above reported results, however, are complicated by the finding that phosphorylation of MDM2 at Ser166 is increased in the absence of Cyclin G1 and decreased in Notch3 depleted cells. Akt and ERK activate MDM2 phosphorylation at Ser166 resulting in diminishing cellular levels and transcriptional activity of p53 [23-25]. However we showed that NC and Notch3 KD cells had very similar expression of p-ERK1/2 and p-Akt [26] (Supplemental Fig. 1B) suggesting that these molecules are not the mechanism through which Notch3 regulates MDM2 phosphorylation at Ser166.

To better investigate the mechanisms associated with the reduced Cyclin G1 protein levels observed in Notch3 depleted cells, we examined Cyclin G1 mRNA expression in the three Notch3 KD HCC cell lines used in this study. We found that Notch3 silencing resulted in increased Cyclin G1 mRNA expression in HepG2 and SNU398 cell lines (Fig.4D). Conversely in Hep3B TP53−/− cells Notch3 depletion resulted in reduced levels of Cyclin G1 transcription (Fig.4D). Taken together these results suggest that Notch3 could regulate Cyclin G1 at transcriptional level leading to decreased Cyclin G1 protein expression in the absence of p53, as suggested by findings obtained in Hep3B cells. On the other hand, the discrepancy between cyclin G1 mRNA and protein modulation following Notch3 silencing in the presence of p53, suggests the prevalence of post-transcriptional mechanisms. The complex regulation of cyclin G1 expression, partly exerted by p53, is well known and can occur at multiple levels [9]. Indeed Cyclin G1 is one of the first identified p53 target gene [9] and p53 transient silencing resulted in decreased Cyclin G1 mRNA levels both in HepG2 and SNU398 cells whereas MDM2 mRNA levels resulted unaffected (Fig.4F). The simultaneous ablation of Notch3 and p53 abrogated Cyclin G1 mRNA induction that is caused by knockdown of Notch3 alone (Fig.4G). These results let us to hypothesize that Notch3 mediates Cyclin G1 down-regulation through transcriptional and post-transcriptional mechanisms in cells harbouring functional p53.

### Notch3 correlates with CyclinG1, MDM2 and p53 expression in human HCC

**Figure 5:**
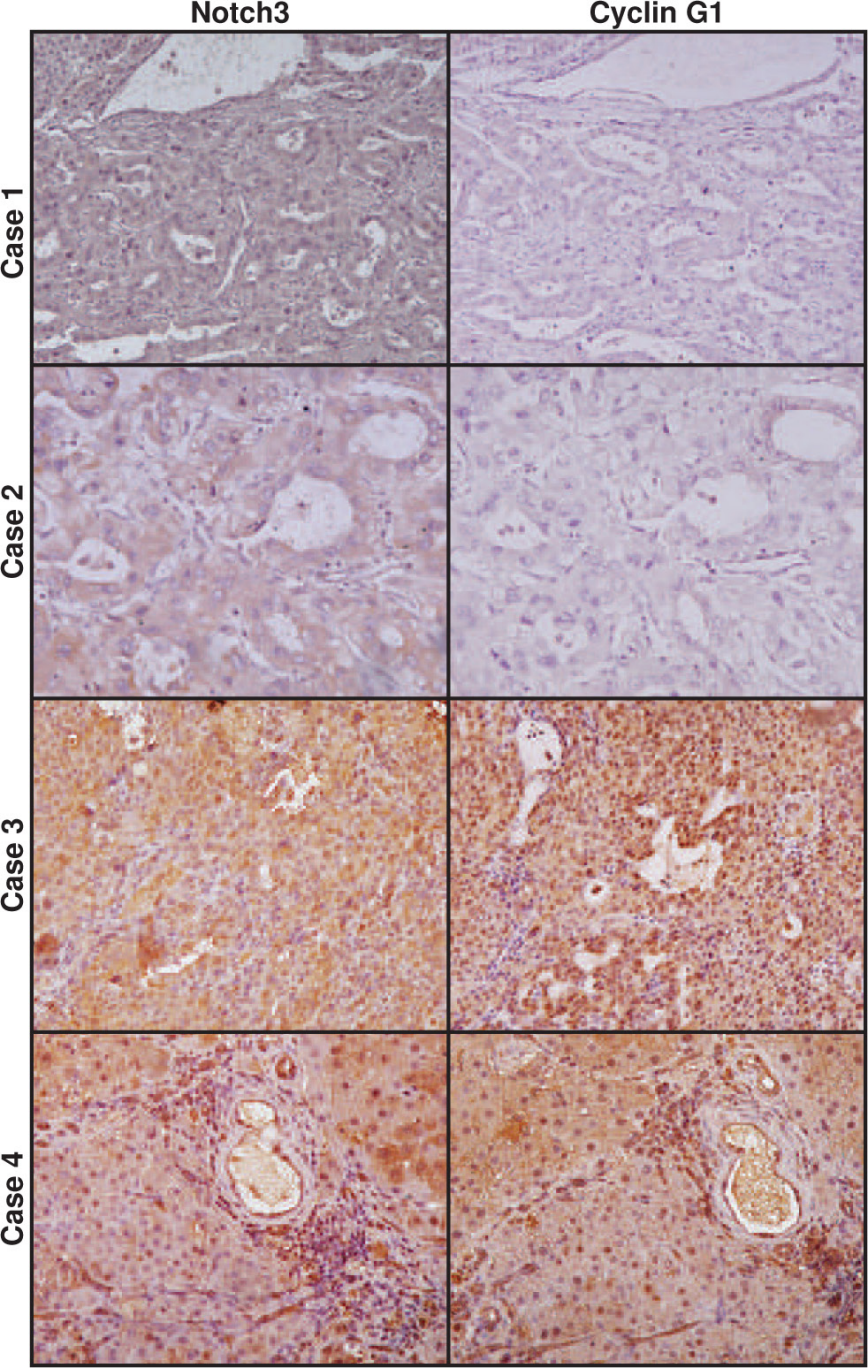
Expression profile of Notch3 and Cyclin G1 in human HCC Immunohistochemistry analysis in four representative cases showing Notch3 and Cyclin G1 expression in the same area. Case 1-2: HCC tissue with Notch3 and Cyclin G1 negative hepatocytes. Case 3-4 show Notch3 and Cyclin G1 staining in the same area. Magnification 20X.

To assess to what extent our in-vitro findings are representative of what occurs in human HCC*, we analyzed the expression of Notch3, Hes1, Cyclin G1 and MDM2 proteins and miR-221 in 27 surgically resected HCCs by western blot and Real-Time PCR respectively (Supplemental Table 1-2). In addition,* the 27 HCC tissues used in the study were analysed for p53 mutations and the correlation between Notch3 and p53 was assessed, using Elisa, only in p53 wild-type samples. A significant inverse correlation was determined between Notch3 and *p53* proteins accumulation (Spearman ρ= −0.575, p<0.05) in 16 patients. *A significant linear correlation was found* between Notch3 and Hes1 (Pearson's correlation, P=0.038) and between Notch3 and MDM2 (Person's correlation, P= 0.005). As expected, no significant correlation was found between Hes1 and miR-221 in the whole HCCs setting. Indeed, we proved that both Hes1 and p53 are involved in miR-221 regulation. Based on this observation we analyzed miR-221 and Hes1 protein expression in cases with mutated p53 and a positive correlation was found (Spearman ρ= 0.709, p<0.05). Finally, a positive correlation was found between Notch3 and *Cyclin G1* (Pearson's correlation, P=0.0063). Since we suggest that p53 accumulation in Notch3 depleted cells is primarily triggered by Cyclin G1 down-regulation *in vitro*, we explored whether Notch3 and Cyclin G1 co-localize in 13 HCCs by immunohistochemistry. Interestingly Notch3 and Cyclin G1 resulted expressed in the same areas and a significant direct correlation between the two proteins accumulation was present (Pearson's correlation, P=0.0062) (Fig.5).

### Notch3 controls Cyclin G1 expression in rat HCCs

**Figure 6:**
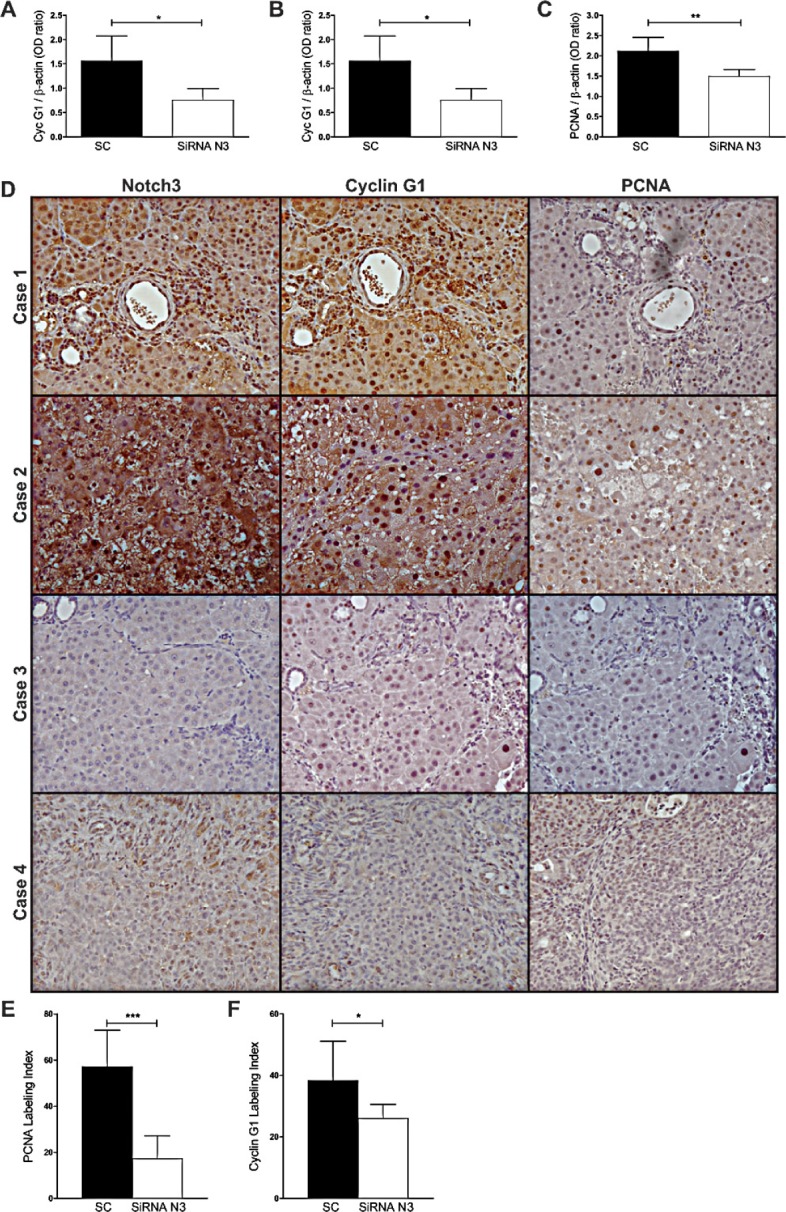
Expression profile of Notch3, Cyclin G1 and PCNA in rat HCCs A-B-C) Notch3, Cyclin G1 and PCNA proteins expression were evaluated in rat HCCs treated with scramble RNA (SC) or with Notch3 siRNAs by western blot. Results are shown as the means of all the analyzed HCCs (+/− S.E.). *, P<0.05; **, P<0.01; P ***, P<0.001 (by two tailed student's t test). D) Immunohistochemistry analysis in representative rats HCC showing Notch3, Cyclin G1 and PCNA expression in the same area. Case 1-2: Rat HCCs treated with scramble RNA; Case 3-4: Rat HCCs treated with Notch3 siRNAs. Magnification 20x. E-F) PCNA and Cyclin G1 were quantified as described in the methods. The numbers were the average of counting 15 fields in each sample. *, P<0.05; ***, P<0.001 (by two tailed student's t test).

To assay whether Notch3 mediated regulation of Cyclin G1 protein expression really occurs in primary tumors we first analyzed Cyclin G1 and Notch3 protein expression in rat HCCs induced by DENA (diethylnitrosamine) and found a positive correlation between the two analyzed proteins (Pearson's correlation, P=0.007) (Supplemental Table 3). Then we analysed HCCs from Notch3 siRNAs injected rats and negative controls. In line with our *in vitro* findings Cyclin G1 protein expression in the HCCs of RNAi group was significantly lower than in the SC group (t-test, P=0.026) suggesting that Notch3 inhibition can modulate Cyclin G1 expression *in vivo* (Fig.6A-B). It has been shown that loss of Cyclin G1 inhibited both the initiation stage and progression of liver cancer induced by DENA [27]. In agreement with this result we observed a direct correlation between Cyclin G1 and PCNA (Pearson's correlation, P=0.0073) and between Notch3 and PCNA (Pearson's correlation, P=0.0070). Immunohistochemistry of Notch3, Cyclin G1 and PCNA on serial sections of the liver from control rats and rats subjected to silencing supports western blot results (Fig.6D-F).

### Notch3 silencing strengthen the effect of MDM2 inhibitors

**Figure 7:**
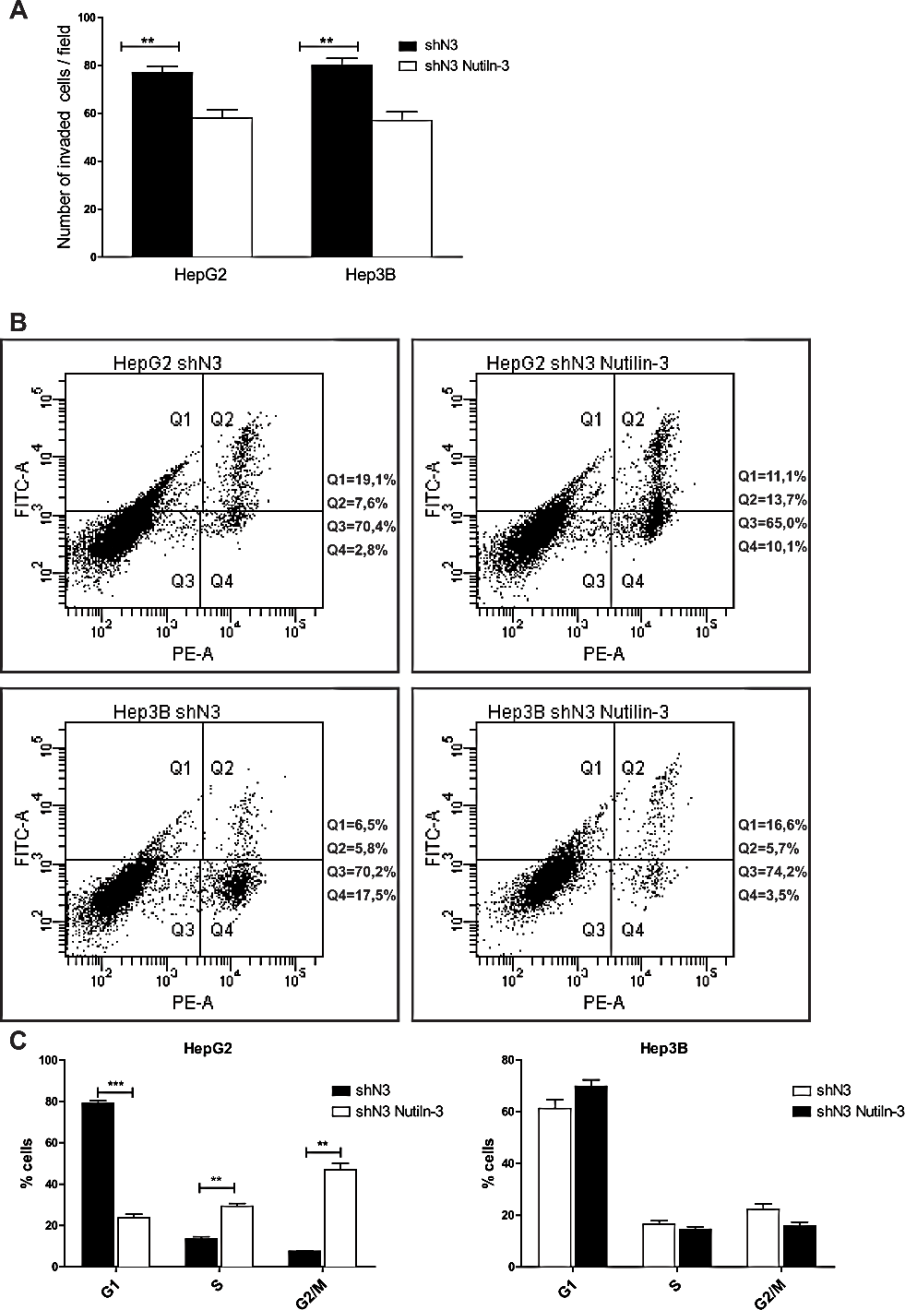
Effects of Notch3 knockdown on Nutilin-3 sensitivity A) Analysis of invasion capacity of Notch3 silenced cells (shN3) treated or untreated with Nutilin-3. B) After treatment with 5μM of Nutilin-3 for 48 h cells were labeled with Annexin V-FITC and propidium iodide. The distribution pattern of live and apoptotic cells was determined by FACS analysis. Viable cells display no Annexin and propidium iodide staining (Q3); early-stage apoptotic cells display high Annexin and low propidium iodide staining (Q1); late-stage apoptotic cells display high Annexin and high propidium iodide staining (Q2); DNA fragmentation is represented by high propidium iodide and low Annexin staining (Q4). X-axis represents propidium staining (PE) and y-axis represents FITC staining. Data are representative of at least three independent experiments. C) Histogram representing the growth rate of HepG2 and Hep3B Notch3 silenced cells (shN3) evaluated by propidium iodide staining and flow cytometry 48h post-Nutilin-3 treatment. Results are the mean of three independent experiments (+/− S.E.) **, P<0.01; ***, P<0.001 (by two tailed student's t test).

The positive correlation between Notch3 and MDM2 observed *in vivo*, suggests that the effect of MDM2 inhibitors can be exacerbated by Notch3 silencing independently by p53 status. Indeed MDM2 has been shown to regulate the expression of proteins that contribute to cell proliferation, apoptosis and invasion independent of its interaction with p53 [28-30]. The treatment of HepG2 and Hep3B Notch3 silenced cells with Nutilin-3 determined reduced cell invasion in both the analyzed cell lines (Fig.7A) and increased apoptosis in Hep3B cells as assayed by Annexin V (Fig.7B). To investigate whether Notch3 silencing combined with Nutilin-3 can affect the distribution through the different phase of the cell cycle FACS analysis was performed. No changes in cell cycle distribution was observed in Hep3B cells treated with Nutilin-3 compared to Notch3 silenced cells (Fig.7C). On the contrary, we observed a significantly decreased in G1 phase population and a corresponding increase in the G2/M phase for HepG2 Notch3 silenced cells treated with Nutilin-3 compared to Notch3 silenced cells (Fig.7C)

## DISCUSSION

Notch receptors are emerging as important players for cancer therapy [2]. Currently, most Notch-directed therapies involve the use of gamma-secretase inhibitors (GSIs). However the use of GSIs is associated with intestinal toxicity in patients, due to the simultaneous inhibition of different proteins [31]. Thus, there is a strong rationale to target the Notch receptors individually. Our previous studies focused on Notch3 in HCC and found that Notch3 activity lies, in part, in the ability of Notch3 to suppress p53 expression [22]. Herein we investigated how Notch3 regulates p53 expression and proposed a post-translation mechanism. We draw this conclusion because we did not observe any change in the mRNA levels of p53 neither following Notch3 inhibition nor upon Notch3 over-expression.

The possibility that p53 stabilization was a consequence of DNA damage activation after Notch3 ablation was ruled out, as we previously showed that control cells had the same endogenous degree of basal DNA damage as Notch3 depleted cells [22]. In line with this we showed that p53(Ser-20) and p53(Ser-15) were not significantly elevated.

Therefore, we focused on different mechanisms that could regulate p53 stability. Stabilization is considered a prerequisite for p53 function and cancer cells often show alterations affecting p53 half-life, such as overexpression of the ubiquitin ligase MDM2 and Cyclin G1 [11].

We show here that the increase in p53 protein expression in Notch3 depleted cells is first mediated by a dramatic decrease of Cyclin G1 in all the analyzed cell lines. Indeed Cyclin G1 inhibition associated with Notch3 silencing abrogated the effect of Notch3 silencing on p53 protein expression suggesting that Notch3 silenced cells regulate p53 protein expression mainly through Ciclin G1.

A positive correlation between Notch3 and Cyclin G1 was confirmed in primary tumors and in HCCs arisen in DENA treated rats. Interestingly Notch3 silencing *in vivo* resulted in reduced levels of Cyclin G1 suggesting a really involvement of Notch3 in Cyclin G1 regulation. Moreover, a strong correlation was evident between Notch3 and PCNA and between Cyclin G1 and PCNA supporting a role of both Notch3 and Cyclin G1 in cancer progression. It has been reported that DENA administration in rat induces a high constitutive expression of MDM2 that attenuates p53 levels [32]. In addition p-MDM2 Ser166 was also induced in rat liver by DENA treatment suggesting a role for pMDM2 Ser166 in p53 degradation [25] in this model. Based on these observation MDM2 and p53 protein expression were not analyzed in rat HCCs upon Notch3 silencing. However, according to *in vitro* results the analysis of Notch3 and p53 protein expression in p53 wild-type HCC revealed an inverse correlation.

It has been reported that Cyclin G1 binds *in vitro* and in vivo to MDM2 and stimulates the ability of PP2A to dephosphorylate MDM2 at Thr216 leading to p53 protein accumulation [12]. In agreement with this observation MDM2 dephosphorylation at Thr216 was observed both in Notch3 and Cyclin G1 silenced cells. On the contrary Notch3 knock down cells display low levels of MDM2 phosphorylated at Ser166 whereas high levels were observed in Cyclin G1 silenced cells compared to negative control. We showed that negative control and Notch3 KD cells have very similar expression of p-ERK1/2 and p-Akt cells, suggesting that these molecules are not associated with the reduced levels of Ser166 phosphorylation warranting future investigation. P53 accumulation in Notch3 depleted cells triggers the up-regulation of its known transcriptional target miR-221 leading to MDM2 reduction and thus to the increased p53 stability. Contrary in Hep3B TP53−/− cells reduced levels of miR-221, in the absence of Notch3 protein, are associated to reduced levels of Hes1 which regulates miR-221 transcription. Furthermore, the combined silencing of Notch3 and p53 in HepG2 p53+/+ cells follows the effects observed in Hep3B, p53-deficient cells. Taken together, our results suggest that the dominant effect on miR-221 transcription is p53 dependent. On the other hand the positive correlation between Notch3 and MDM2 observed *in vivo*, suggests that the effect of MDM2 inhibitors can be strengthened by Notch3 silencing independently by p53 status. In agreement with the latter, we showed that Notch3 ablation exacerbated the response to Nutilin-3 affecting invasion. Moreover, loss of Notch3 in Hep3B resulted in their enhanced Nutilin-3 mediated death as revealed by Annexin V-FITC staining. In agreement with previous studies, upon exposure to Nutilin-3, Notch3 KD HepG2 cells accumulate in the G2/M phase of cell cycle [33]. These data indicate that Nutilin-3 induces cells to accumulate in G2/M without significant cell death in the presence of wild-type p53.

**Figure 8:**
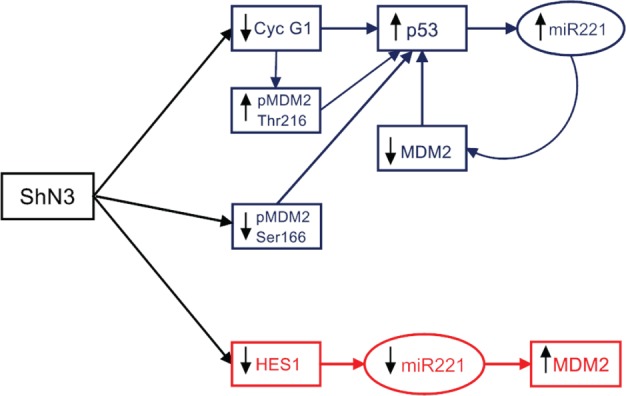
Notch3 regulates p53 expression in tumour cells Blue panel: increased in p53 protein expression is triggered by Cyclin G1 and sustained by the miR-221-MDM2 axis in p53 wild Type HCC cells upon Notch3 inhibition. Red panel: Hes1 regulates miR221 expression and thus MDM2 levels in Hep3B cells (p53−/−).

Our observations let us suppose that the combination of Notch3 silencing with MDM2 inhibitors might induce a stronger response in HCC treatment by mediating a “Horizontal blockade”. Future studies will be directed towards dissecting the mechanism for this novel Notch3-MDM2 crosstalk in HCC warranting *in vivo* studies.

Finally, no correlation was found between Notch3 and miR-221 and between MDM2 and miR-221 in our human HCCs setting, supporting the hypothesis, that miR-221 is not the only actor in MDM2 regulation *in vivo*. The results add further support for the growing evidence of the usefulness of Notch3 as a therapeutic target for hepatocellular carcinoma.

## MATERIAL AND METHODS

### Ethics Statement

Investigation has been conduced in accordance with the ethical standards and according to the Declaration of Helsenki and according to national and international guidelines and has been approved by the authors institutional reviewed board.

### Cell lines and Gene silencing by retroviral transduction of shRNAs

The human hepatocarcinoma cell lines HepG2, SNU398 and Hep3B were obtained from American Type Culture Collection (ATCC, Rockville, MD, USA). HepG2 and Hep3B cells were maintained in Eagle's Minimum Essential Media (MEM) while SNU398 cells were maintained in RPMI. Media were supplemented with 10% of fetal bovine serum (FBS), 100 U/ml of penicillin, and 100 mg/ml of streptomycin (all reagent from ATCC) at 37 °C in 5% CO_2_ incubator. Notch3 and Cyclin G1 knock down (KD) were obtained using short hairpin oligonucleotides targeted to different exons inserted into the pSuper.puro expression vector (OligoEngine, Seattle, WA) as previously described [22]. Since two Notch3 and Cyclin G1 specific shRNAs were equally effective in our previous study [22, 34] we performed the experiments by selecting single shRNAs. Cells harbouring a pSuper.puro provirus expressing a GL2 luciferase specific shRNA were used as negative control (NC) [35]. ShOligos targeted to different *Hes1* exons were purchased from Origene (OriGene Technologies Inc., Rockville, MD) and their efficacy was evaluated as previously described [18].

### SDS-PAGE and Western blot analysis

Protein extraction and immunoblotting were performed as previously described [36]. Primary antibodies were as follows: anti-Notch3 polyclonal antibody (sc-5593, Santa Cruz Biotechnology, Santa Cruz, CA, USA), anti-p21 monoclonal antibody (Clone SX118, Dako, Denmark), anti-p53 monoclonal antibody (Clone DO-7, Dako), anti-phospho-MDM2 Ser166 polyclonal antibody (3521, Cell Signaling Technology, Beverly, MA), anti-MDM2 (SMP14) monoclonal antibody (sc-965, Santa Cruz Biotechnology), anti-MDM2 (N-20) polyclonal antibody (sc-813, Santa Cruz Biotechnology) anti-Cyclin G1 monoclonal antibody (sc-7291, Santa Cruz Biotechnology), anti-pAkt monoclonal antibody (2965, Cell Signaling), anti-Akt polyclonal antibody (209020, Cell Signaling), anti-pERK monoclonal antibody (sc-7383 Santa Cruz Biotechnology), anti-ERK polyclonal antibody (9102, Cell Signaling), anti-phospho p53 ser 20 polyclonal antibody (sc-18079-R Santa Cruz Biotechnology), anti-phospho p53 ser15 polyclonal antibody (9284 Cell Signaling Technology) and anti-β-actin monoclonal antibody (Clone AC-40, Sigma, ST Louis, USA) Immunoreactivities were revealed with the EnVision dextran polymer visualization system (Dako). Membranes were washed and autoradiographies were obtained using a chemiluminescence reaction (ECL reagents, Amersham) Digital images of autoradiographies were acquired with a scanner (Fluor-S MultiImager, Bio-Rad) and signals were acquired in the linear range of the scanner and quantified using a specific densitometric software (QUANTITY-ONE, Bio-Rad) in absorbance units.

Expression vector. The active form of Notch-3, pNICD-3, was cloned by PCR with forward (TCTTGCTGCTGGTCATTCTC) and reverse (GGCCCCCAAGATCTAAGAAC) primers using Herculase Taq polymerase (Stratagene, La Jolla, CA). The PCR product was inserted into pcDNA3.1/V5-His Topo TA Expression Vector (Invitrogen).

### Transfections

HepG2 cells were seeded into 6 well plates and transfected with 20 nM of p53 (Invitrogen), Cyclin G1 (IDT, Thief River Falls, MN, USA) or scrambled siRNA (scRNA) using Lipofectamine 2000 (InVitrogen). Transfection efficiencies were greater than 90% as determined by co-transfection with a fluorescein-labelled siRNA (InVitrogen). For plasmid transfection cells were seeded into 6 well plates and transfected with 0,2 μg of pNICD-3 plasmid or empty vector using Lipofectamine 2000. Analysis of genes and proteins expression were performed 24h and 48h post transfection.

### Cyclin G1 and Notch3 double silencing

Stable, retroviral transduced populations of HepG2 cells (NC and shG1) were selected in growth media supplement with Puromycin. Once selected, Negative Control (NC) and Cyclin G1 (shG1) silenced cells were transfected with a pool of Notch3 siRNAs (OriGene, MD, USA) using Lipofectamine 2000 (InVitrogen). Evaluation of proteins expression was performed 48h post-transfection by western blot.

### RNA analysis

Total cellular RNAs were prepared with Trizol (Invitrogen) according to the manufacturer's instructions. One microgram of total RNA were reverse-transcribed using Superscript II (Invitrogen). Relative gene expressions were determined by semi-quantitative end-point PCR. PCR primers were reported in Table 1.

**Table 1:** Primer sequences for RT-PCR and ChIP analysis

Gene	Primers sequence (5′-3′)	Annealing T (°C)	Cycle n°	Product size (bp)*	Analysis
MDM2 F† MDM2 R ‡	GAGCCTCCAATGAGAGCAAC AGGCTGCCATGTGACCTAAG	61	31	87	RT-PCR
Cyclin G1 F Cyclin G1 R	AATGAAGGTACAGCCCAAGCA GCTTTGACTTTCCAACACACC	63	27	197	RT-PCR
P53 F P53 R	GGCCCACTTCACCGTACTAA GTGGTTTCAAGGCCAGATGT	57	29	150	RT-PCR
β ACTIN F β ACTIN R	gaggcactcttccagccttc ggatgtccacgtcacacttc	55	26	189	RT-PCR
P21 F P21 R	GGAGACAGGAGACCTCTAAAGACC ACACAAGCACACATGCATCA	63	35	119	ChIP
MiR-221-S1 F MiR-221-S1 R	aagctggatggaaggaaggt ccatccacccatttatccat	63	40	99	ChIP
MiR 221-S2 F MiR 221-S2R	ttcatttatccaccccagaaa tttcagtctttttctaccctttcc	60	40	168	ChiP
MiR 221-S3 F MiR 221-S3 R	catgaccacatggccaatta tagctgcatgtccgatcaaa	60	40	195	ChiP

* bp, base pairs

† F, forward

‡ R, reverse

### Real-Time PCR

*MiR-221*, was assessed by using Taq-Man MicroRNA Assays (Applied Biosystems, Foster City, CA, USA), as previously described [38].

### FACS analysis

Stably infected cell populations of HepG2 and Hep3B were seeded into 6-well dishes and allowed to attach for 24 hours before treatment with 5um of Nutilin-3 (Sigma). Apoptosis was revealed by Annexin V-FITC (Bender Medsystems, Vienna, Austria) staining 48h post Nutilin-3 treatment with Fluorescent-Activated Cell Sorter (BD FACSaria cell sorter, BD Bioscences, San jose, CA, USA. Parenthesis cells were collected, washed twice with PBS, fixed with 70% cold ethanol at −20°C, resuspended in 500 μl of PBS containing 10 μg/ml propidium iodide and 50 μg/ml RNase A and incubated for 30 min at room temperature. Cells were then centrifugated at 1200 rpm for 5 min, resuspended in PBS and analyzed with FACS.

### Cell invasion assay

Cell invasion was assessed by Boyden blind-well chambers containing poly-vinyl-pyrrolidone–free polycarbonate filters, 8-μm pore size coated with Matrigel (Sigma). Twenty-four hours after the Nutilin-3 treatment, 5.0 × 10^4^ HepG2 and 3.0 × 10^4^ Hep3B cells were resuspended in serum-free medium and added to the upper chamber. A medium supplemented with 30% FBS was used as chemoattractant to the lower chamber. After 24h of incubation, non invading cells were removed from the upper surface of the filter with cotton swabs. Invasive cells were fixed with 4% paraformaldehyde, stained with Giemsa (Sigma), and counted under a microscope.

### *In vivo* Notch3 silencing

Male Wistar rats (Harlan, Udine, Italy) were used in the study. All animals received human care in accordance to the criteria prepared by the National Academy of Sciences and published by the National Institutes of Health (NIH publication 86-23 revised 1985). All protocols were approved by local ethic committee. DENA was given in the drinking water (100 mg/l) for 8 weeks [38]. *In vivo* delivery of siRNA was performed using 4 ug of siRNA (Sigma) per gram of body weight. Seven rats were included in each groups (SC and SiRNA N3). SiRNAs were injected every 3 days into the tail vein. For tail-vein injection siRNA was applied in a total volume of 0.5 ml (0.25 ml of PBS, 0.25 ml *In Vivo* RNA-LANCEr, Sigma). Rats were sacrificed at day 12 and freshly harvested HCCs were subjected to protein lyses and analyzed by western blot.

### Patient samples

Twenty-seven patients of both sexes undergoing partial hepatectomy for HCC entered the study. Informed consent was obtained from each patient according to Italian guidelines and the latest version of the Helsinki Declaration. Exclusion criteria were a previous history of local or systemic treatments for HCC. Tissues sample were fixed in 10% formalin and paraffin-embedded for histopathology and immunohistochemistry.

### TP53 mutation

Exons 4–10, along with flanking intronic boundaries of the *TP53* gene [GenBank Reference No. NC_000017.10 (7571720-7590863); RefSeqGeneID NG_017013.1] were screened by WAVE denatured high-performance liquid chromatography (dHPLC) instrument (Transgenomic, San Jose, CA, USA). PCR products showing the presence of heteroduplexes were directly sequenced to characterize nucleotide variants on the ABI PRISM 3730 Genetic Analyzer (Applied Biosystems), using standard protocols.

### Immunohistochemistry (IHC)

The presence and localization of Notch3, Cyclin G1 and PCNA in HCCs, were immunohistochemically assessed on formalin-fixed, paraffin-embedded sections. Serial 4 μm thick sections were processed for haematoxylin and eosin staining and for immunohistochemistry. Endogenous peroxidases were inhibited by incubating slides in 3% H_2_O_2_–methanol for 20 min at 4°C. For antigen retrieval, slides were immersed in pH 6.0 citrate buffer (pH 6.0) and boiled using a microwave owen. Negative controls were obtained by omitting the primary antibody. Immunoreactivity was revealed with the EnVision system (DAKO), and diaminobenzidine (DAB) as chromogen (Sigma). Slides were counterstained in Meyer's haematoxylin, coverslipped and examined by light microscopy. Hepatocellular carcinomas were categorized according to nuclear, membranous and cytoplasmic Notch3 immunostaining. Staining of sections was assessed on 15 consecutive 40X magnification fields by two independent observers (L. G., C. G.) using a validated semi-quantitative scale where 0, absence of staining; 1, staining of 5%-30% of hepatocytes; 2, staining on > 30% hepatocytes. Cyclin G1 and PCNA staining were quantified by image cytometry using Image J software (NIH, Bethesda, USA) on at least 15 randomly selected consecutive fields at 40X and expressed as the percentage of positive nuclei over the total nuclei (Labeling index:LI). Results represent the average of the percentage from 15 consecutive 40X magnification fields.

### Statistical analysis

Differences between groups were analyzed using a double-sided Student t-test. Experimental data are expressed as the mean ± SE from three independent experiments. Pearson's correlation was used to explore the relationships between Notch3 and Hes1, between Notch3 and MDM2, between Notch3 and Cyclin G1, between Notch3 and PCNA and between Cyclin G1 and PCNA expression in HCC tissues. Spearman's correlation was used to explore the relationships between Notch3 and p53 or miR-221 and Hes1 expression in HCC tissues. P-values less than 0.05 were considered statistically significant. Statistical analysis were performed using SPSS version 19.0.

## SUPPLEMENTARY MATERIAL FIGURE AND TABLES


