# Supplementary material for: Suppression of p53 by Notch3 is mediated by Cyclin G1 and sustained by MDM2 and miR-221 axis in hepatocellular carcinoma
Source: Oncotarget. 2014 Sep 25;5(21):10607–20. doi: 10.18632/oncotarget.2523 (PMC4279397; doi:10.18632/oncotarget.2523)
Supplement: Supplementary file 1 [file oncotarget-05-10607-s001.pdf]

Suppression of p53 by Notch3 is mediated by Cyclin G1 and sustained by MDM2 and miR-221 axis in hepatocellular carcinoma

Supplementary Material

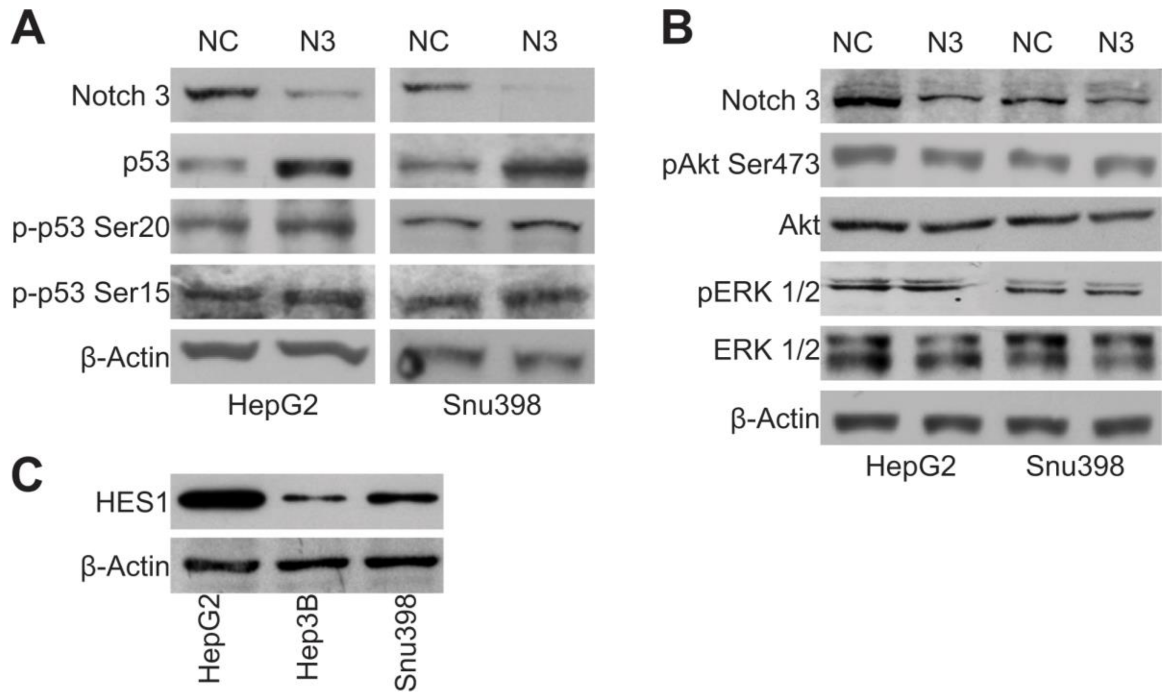

Supplemental Figure 1: Specific proteins expression. (A, B) p-p53(Ser20), p-p53(Ser-15), pAKT(Ser473), AKT, pERK1/2 and ERK were evaluated by western blot in HepG2 and SNU398 Notch3 silenced cells. NC: shRNA; N3; Notch3 shRNA. (C) Hes1 protein expression was evaluated by western blot in the three HCC cell lines used in the study.

Supplemental Table 1.

Characteristics of HCC patients

Gender (M/F)	20/7
Age (median±SD)	67.2±7.9
Etiology of CLD	
HBV (%)	6/27 (22.2%)
HCV (%)	17/27 (63%)
None (%)	4/27 (14.8%)
Focality	
Uni-focal (%)	17/27 (63%)
Multi-focal (%)	10/27 (37%)
AFP	
< 20 ng/mL (%)	17/27 (63%)
> 20 ng/mL (%)	10/27 (37%)
Grading	
G1 (%)	1/27 (3.8%)
G2 (%)	6/27 (22.2%)
G3 (%)	15/27 (55,5%)
G4 (%)	5/27 (18.5%)

Supplemental Table 2: Proteins and miR-221 expression in 27 HCC specimens.

Patient	Notch3	Cyclin G1	MDM2	Hes1	miR-221
1	0,05	1,03	0,13	1,74	
2	0,11	3,71	11,13	6,37	0,1
3	0,02	0	0,92	1,06	0,92
4	0	0	2,93	0,03	1,04
5	0,07	0	0,1	1,52	0,4
6	0,12	0	2,13	0,59	0,72
7	0,12	0	3,91	1,43	
8	0,08	0,18	4,1	0,7	0,95
9	0,14	0	0	2,51	0,35
10	0,72	6,68	8,84	3,04	0,7
11	0,41	1,08	2,93	1,7	0,58
12	0,05	1,54	0,52	2,69	2,37
13	0,07	1,12	2,21	0,2	0,13
14	0,09	0,8	0,79	2,72	0,27
15	0	0	0,35	0,02	
16	0,11	5,14	2,54	1,27	
17	0,19	2,62	0,45	2,64	0,49
18	0,16	0,55	9,34	0,08	0,3
19	0,32	0,67	4,83	2,61	
20	0,35	5,63	26,2	2	0,44
21	0,24	0	19,7	1,5	
22	0	0,67	0,76	0	0,39
23	0	0,95	1,25	0,94	0,59
24	0,07	1,04	1,54	2	0,5
25	0,08	0	1,25	0,1	0,31
26	0,01	0,39	0,54	0,1	1,45
27	0,02	0	1,45	0	0,39

	Notch3	Cyclin G1	MDM2	Hes1	miR-221
Notch3 Pearson's Correlation<br>P value		P=0.0063	P=0.005	P=0.038	
Hes1 Correlation Coefficient<br>P value	P=0.038				P=0.086

Supplemental Table 3: Proteins expression in 11 rat HCC specimens

Rat	Notch3	Cyclin G1
1	0,34	1,13
2	0,97	4,74
3	0,48	2,53
4	0,29	0,36
5	0,25	1,32
6	0,37	0,44
7	0,37	1,65
8	0,42	2,22
9	0,44	1,45
10	0,10	1,54
11	0,14	2,7

	Cyclin G1
Notch3 Pearson's Correlation<br>P value	P=0.007
